# The seed water content as a time-independent physiological trait during germination in wild tree species such as *Ceiba aesculifolia*

**DOI:** 10.1038/s41598-020-66759-3

**Published:** 2020-06-26

**Authors:** Ximena Gómez-Maqueo, Diana Soriano, Noé Velázquez-Rosas, Sandra Alvarado-López, Karina Jiménez-Durán, María del Mar Garciadiego, Alicia Gamboa-deBuen

**Affiliations:** 1 Instituto de Ecología, Universidad Nacional Autónoma de México (UNAM), Mexico City, México; 2 Instituto de Biología, UNAM, Mexico City, Mexico; 3 Centro de Investigaciones Tropicales, Universidad Veracruzana, Veracruz, Mexico; 4 Facultad de Química, UNAM, Mexico City, Mexico

**Keywords:** Ecology, Molecular biology, Plant sciences

## Abstract

Seeds constitute a key physiological stage in plants life cycle. During seed germination, there is a spatial-temporal imbibition pattern that correlates with described physiological processes. However, only the moment of testa rupture has been described as a critical, discrete stage. Could a specific relative water content (RWC) value reflect a physiological stage useful for comparisons between seed batches? We tracked seed-by-seed imbibition during germination to homogenize sampling and selected a transcriptomic approach to analyse the physiological transitions that occur in seed batches collected in different years and with contrasting phenotypic responses to a priming treatment. The seed RWC reflected the transcriptional transitions that occur during germination, regardless of imbibition time or collection year, and revealed a set of biological processes that occur in the dry seed and during early germination are associated with the phenotypic response to priming. As climate shifts, so do the timing of developmental events important for determining organismal fitness, and poses another challenge to the comprehension of molecular and physiological processes driving the interaction between organisms and environment. In this study, we demonstrate that the use of physiological traits, specific to a particular developmental stage, is a reliable time-independent approach.

## Introduction

Seeds constitute a key physiological stage in plants life cycle, and play a critical role in plant communities^1^. At the molecular and genetic level, seed germination in wild species is a field scarcely explored; most of the basic mechanisms driving seed germination and dormancy alleviation have been described in crops and model species. Domestication has had an important impact on genetic diversity altering seed traits that regulate germination^2,3^. Wild species retain the genetic diversity, reflected in a broader spectrum of physiological responses to the environment. Additionally, the environmental conditions during seed development and maturation also have an important effect on seed germination or storability capabilities^4^. Both situations hamper the establishment of appropriate comparison groups in wild plants.

Seed water status regulation plays a major role trough every stage of development, maturation, shedding, and germination^5,6^. Germination begins with water uptake, which triggers in a progressive way the cellular processes needed for radicle protrusion^7^. A highly conserved spatial-temporal imbibition pattern during seed germination was confirmed using Nuclear Magnetic Resonance in tobacco^8^, wheat^9^, and *Brassica rapa*^10^ that correlates with previously described physiological processes during germination, suggesting that the imbibition dynamic could be useful to select specific stages for comparison among seed batches. Imbibition also triggers important transcriptomic changes, as described in several species like rice^11^, *Lepidium sativum*^12^, *Arabidopsis*^13–15^, and wheat^16^. Dekkers *et al*.^13^ described two transcriptomic phases that distinguish “early germination” processes, associated with the onset of repair mechanisms and protein synthesis, and a removal of genes from the previous developmental programs; the “late germination” phase coincides with the moment of testa rupture, a fundamental physiological stage in endospermic seeds, and an enrichment of processes related to metabolism and reserve mobilization required for post-germination establishment. These discrete, well characterized physiological stages for sample collection instead of time-specific collection points provides with more homogeneous biological replicates^13,17^.

Seed-water relations are fundamental in ecosystems with marked seasonality, where seeds are exposed to hydration-dehydration cycles due to stochastic rains, before the actual rainy season sets in, termed as natural priming^18,19^. Like natural priming, the diverse priming treatments performed under controlled conditions can improve germination and optimize field management of many crop species^20^. Here, we characterize the germination process and response to priming across different seed batches collected in different years, from the same population of the wild tree *Ceiba aesculifolia* (*Malvaceae*). This species is resistant to the environmental conditions common in disturbed ecosystems by human activity, such as higher exposure to sunlight, and poor, shallow soils with low water retention^21,22^, and produces “kapok” fibres with potential industrial uses^23^. It has relatively large, orthodox seeds, allowing for individual seed tracking of the relative water content (RWC) during imbibition. Could a specific RWC value reflect a physiological stage useful for comparisons between seed batches and phenotypic responses to priming? We selected a transcriptomic analysis approach to answer this question.

## Results and discussion

### Characteristics of *Ceiba aesculifolia* seeds and germination performance

**Figure 1:**
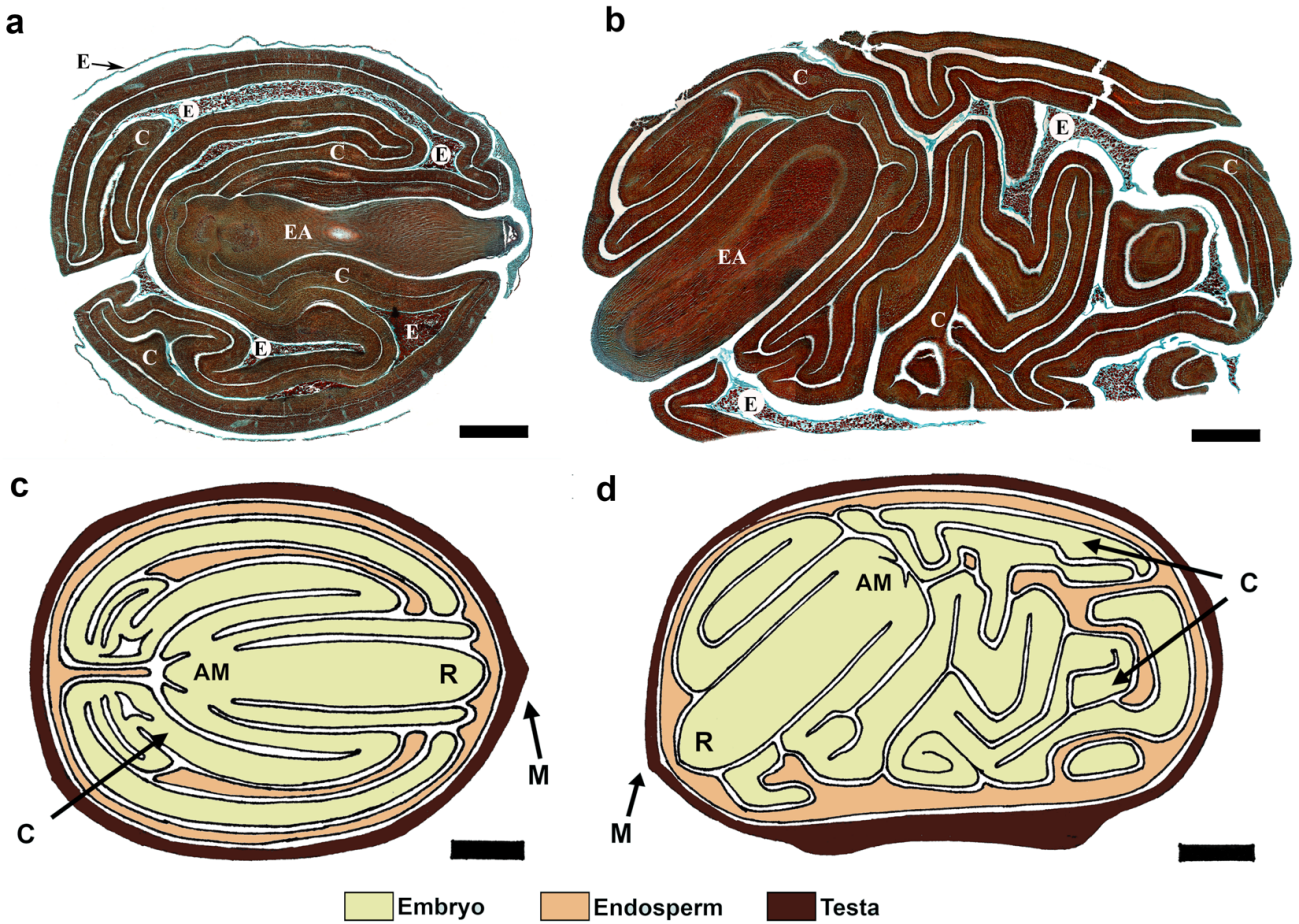
Seed anatomy. In (**a**) transversal, and (**b**) longitudinal sections (10 µm thickness) showing the embryo and remnants of endospermic tissue surrounding the embryo and allocated within the folds of the cotyledons. The sections were stained with Safranin-green fast^37^. EA, embryonary axis; C, cotyledons; E, endosperm. Section scale denoted by the black bars (1 mm). In (**c**) transversal, and (**d**) longitudinal diagrams depicting the full structure of the seed, with the embryo (light green) surrounded by the endosperm (orange), and the testa (brown). AM: apical meristem; C: cotyledons; M: micropyle area; R: radicle tip.

The *C. aesculifolia* seeds present an embryo with large and tightly folded cotyledons, which comprises the majority of the seeds volume (Fig. 1). The testa was removed for microtome sections in Fig. 1a,b. A schematic representation of the whole seed is presented in Fig. 1c,d. The mature seed presents a thin layer of endosperm surrounding the whole embryo that thickens near the micropyle area, where the radicle tip is located. However, the endosperm is not entirely consumed and remnants of endospermic tissue can be found enclosed within the folds of the cotyledons.

*Ceiba aesculifolia* thrives in dry tropical forests that have a stationary rainy pattern, and seeds are released at least four months before the rainy season (Supplementary Fig. S1). However, during the dry period, occasional rains can occur and the seeds are subjected to hydration/dehydration cycles that can be considered as natural priming since no germination occurs at that moment^18,19,24^. In order to study the response to these cycles on transcriptional processes, we determined two phenotypes related to the priming response. The first phenotype corresponds to seed batches that have a positive response to priming (PR, determined as an improvement of the germination parameters tested). The non-responsive/negative phenotype (NR) was comprised by the batches that had no response to priming (2012-5 y and 2014-3 y) and the 2016 batch, which had a negative response to priming by displaying a significant reduction in the germination rate and a slight decrease in the final germination percentage (Supplementary Table S1). Three independent batches were considered for each category. The priming test was performed as a means to classify the seed batches, thus the transcriptomic analyses and results presented correspond only to untreated (control) seeds.

**Figure 2:**
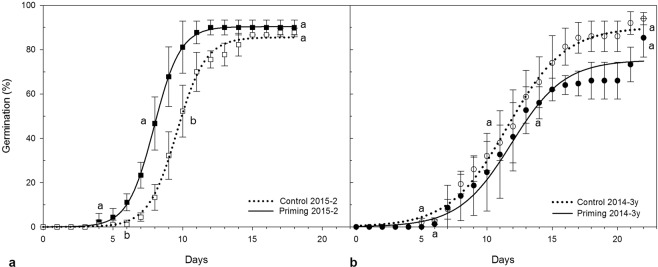
Seed germination performance of two seed batches that represent the studied phenotypes. Doted lines indicate the germination dynamic of the control (untreated) seeds, while bold lines denote the primed seeds. In (**a**) seeds collected in 2015, (**b**) seeds collected in 2014 and stored for 3 years. The letters indicate differences found between primed and control seeds for germination initiation (letters near the x axis), time to 50% (letters at the middle of the sigmoid curves), and final germination (letters near the top end of the curve).

The PR phenotype was represented by one seed batch from 2014 (named 2014) and two 2015 batches from round fruits (2015-1) and elongated fruits (2015-2) from different trees (Fig. 2 and Supplementary Fig. S2). In the untreated seeds, radicle protrusion started between day 5 and 7 after sowing, and took up to 20 days for the seed batch to attain its maximum germination percentage, while primed seeds started at day four in all seed batches. In all cases, un-germinated seeds (about 8 to 15% of seeds in each replicate) died within a 25-day timeframe due to microorganism infection. The positive response to the priming treatment was reflected in either a reduction of the time needed for germination initiation (at day four after sowing) or the time to attain 50% germination, which varied among seed batches. The primed seeds from the 2014 seed batch had a higher maximum germination rate compared to its respective control, while the 2015-1 seed batch was the only batch that showed a significantly slower rate in comparison to its respective control (2014: control 8.95%·day^−1^, primed 10.65%·day^−1^ *P* = 0.02; 2015-1: control 12.4%·day^−1^, primed: 7.96%·day^−1^ *P* = 0.03; 2015-2: control 13.08%·day^−1^, primed 14.69%·day^−1^, *P* = 0.31). There was no significant effect on the final germination percentage except for the 2014 seed batch (2014: control 86.45%, primed 92.33%, *P* = 0.03; 2015-1: control 87.78%, primed 85.56%, *P* = 0.34; 2015-2: control 87.78%, primed 90%, *P* = 0.37). Full measurements are presented in Supplementary Table S1.

The NR phenotype was represented by two batches that were stored for 5 years (2012-5 y) and 3 years (2014-3 y), and one batch recently collected from trees with elongated fruits (2016). Figure 2 and Supplementary Fig. S2 show that there is no effect of priming on the germination parameters for these batches, except for germination rate in 2016 primed seeds, which was significantly lower than its control (Supplementary Table S1). Final germination percentage of 2012-5 y (71.43%) and 2014-3 y (94.0%) batches were similar to the germination performance of those seed batches when freshly collected and had a positive response to priming (80% and 86.45%, respectively). These results suggest that, although these batches had lost the positive response priming, storage has not had a deleterious effect on germination capacity. The 2016 seed batch displays the lowest germination capacity at 53.33%, but is not the result of storage-mediated deterioration or from the induction of secondary dormancy, but rather from a higher mortality during imbibition. This higher mortality could be the result of stress experienced by the mother plants during seed production and maturation. The 2016 batch is different from all other batches (both NR and PR) due to a change in the phenology in that year, the un-timely flowering season in turn caused an early release of the mature seeds on December 2015, two months before its regular season.

## The imbibition dynamic of *C. aesculifolia* seeds is not affected neither by collection year nor response to priming

### Seed imbibition dynamic follows a specific pattern that correlates to the seed RWC

There is a species-specific basal seed water content that functions as a threshold that needs to be exceeded in order for radicle protrusion to occur. This was observed in some domesticated plant species, like cotton, sorghum, chickpea, and maize^25^. Consequently, we asked whether there are other critical thresholds along the imbibition curve. We tracked seed imbibition during the germination process to test this hypothesis.

**Figure 3:**
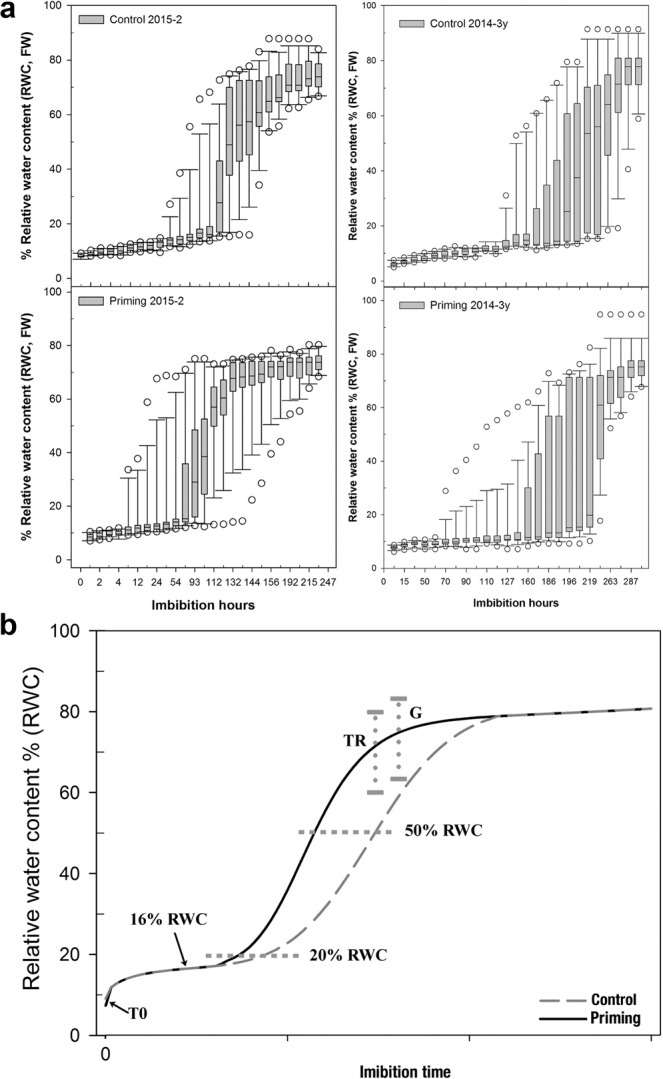
Seed imbibition dynamic. In (**a**) boxplot graphs depicting the variation of seed RWC over a specific time of measurement of the seed batches of 2015-2 (left) and 2014-3 y (right): the RWC dispersion by time of measurement in the control seeds (upper) and the RWC dispersion by time of measurement in primed seeds (lower). Note: The X axis is represented as a category rather than a continuous scale to allow the even distribution of all boxes. In (**b**) a general imbibition dynamic model proposed for control and primed *C. aesculifolia* seeds. Testa rupture (TR) can be most frequently observed when the seed has attained a RWC between 72–85%. Radicle protrusion (G) can occur almost simultaneously to TR, so its RWC interval (75–93%) overlaps with TR.

The variation of seed RWC over a specific time of measurement on both PR- and NR-batches is shown Fig. 3a and Supplementary Figs. S3 and S4. A randomized sampling as a function of time could lead to a mixture of different imbibition or physiological stages. However, by tracking seed-by-seed changes in fresh weight, a similar pattern of imbibition can be observed, which is summarized in Fig. 3b. The seeds have an initial RWC of 7–8% (T0, or the dry state), and imbibe rapidly until they almost double their RWC. At this point, the rate of water uptake slows down; the duration of this phase appreciably varied between seed batches, and treatments. The next RWC value at which the imbibition rate changed again coincided with a 20%RWC. The priming treatment decreased the average imbibition time needed to reach the 20%RWC. The next imbibition phase showed the major change in seed fresh weight. Although there was not a specific RWC value at which all seeds presented testa rupture (TR) or radicle protrusion (G), the RWC interval at which both processes occurred was similar in all seed batches.

In order to establish a physiological trait that allow the comparison between seeds from different batches or treatments for molecular studies, we determined to collect the seeds of each category and batches before TR based on the RWC value for the transcriptional analyses. For PR-batches, we selected 16%RWC as the representative value for the slow imbibition phase and 50%RWC for the rapid imbibition phase. The 50%RWC was selected as the representative value for the early germination phase in the NR batches.

To explore the mechanism underlying the differences in priming responses of the two categories of *C. aesculifolia* seed batches, transcriptome analyses of each phenotype during germination were performed using Illumina clean reads for *de novo* transcriptome assembly. Considering that the germination process involves an important removal of stored transcripts related to seed development, we used only the transcripts with a complete coding sequence (see methods) for further analyses. A total of 54,793 complete transcripts was obtained, which then were identified using a local data base that included three annotated species from the *Malvaceae* family and *Arabidopsis*. We were able to associate a specific *Arabidopsis* locus-tag to 91% of the transcripts, which were used for GO-term enrichment of biological functions.

## The RWC is a physiological trait that reflects the transcriptional transitions that occur during germination

### The *C. aesculifolia* seed batches with positive response to priming present the two main transcriptional phases, as described for Arabidopsis: early and late germination

**Figure 4:**
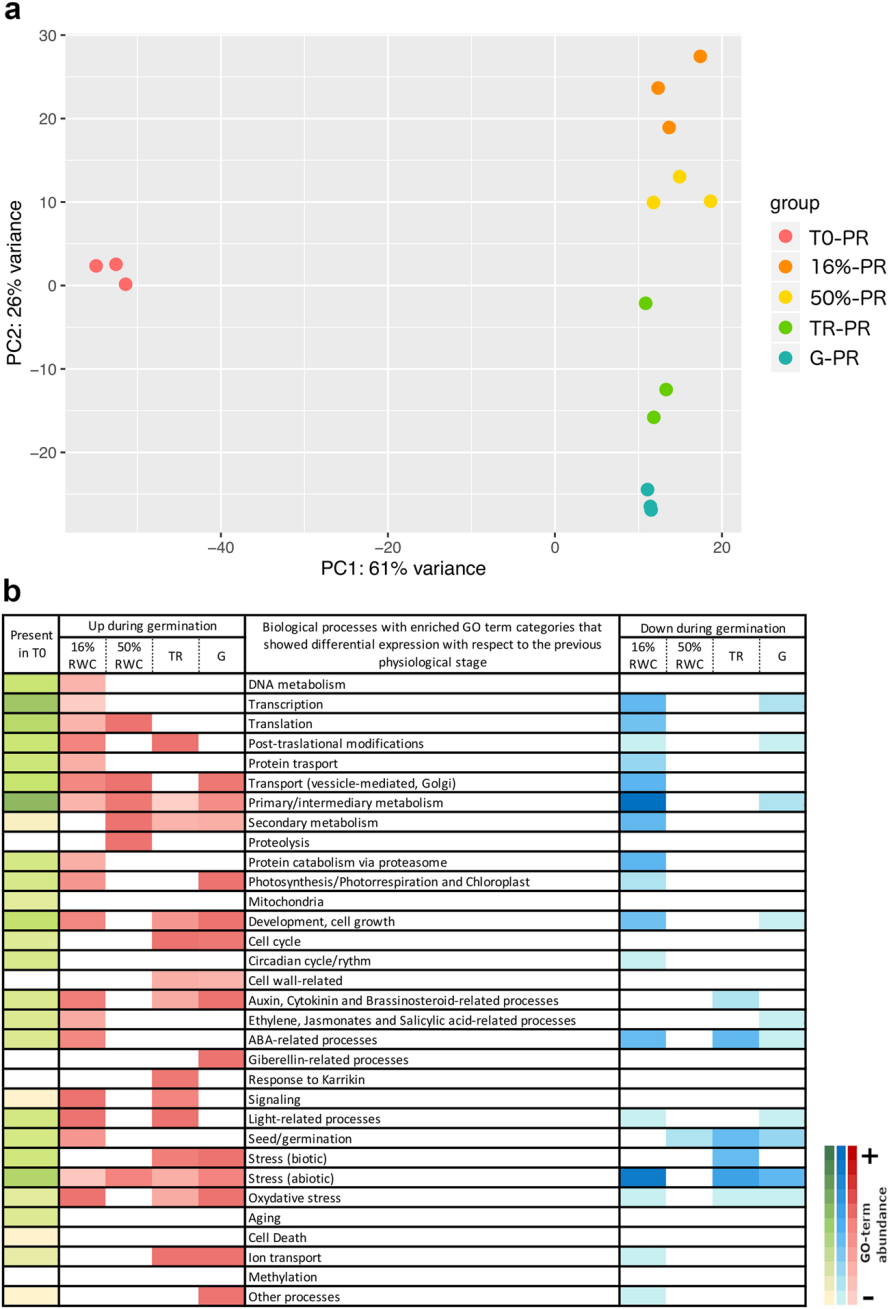
Transcriptome analyses of seed batches with a positive response to priming. In (**a**) PCA analysis of the transcriptome profiles of three replicates for each physiological stage indicated by colour. PC1 explains 61% of the variation between the dry stage and the imbibed stages. PC2 explains 26% of the variation between imbibed stages. In (**b**) Main GO-term categories enriched in each physiological stage. The green colour represents the enriched categories present in the dry seed while the red and blue colours represent the enriched categories that had differential expression with respect to the previous stage. Up-regulated categories are shown in red and down-regulated categories in blue.

**Figure 5:**
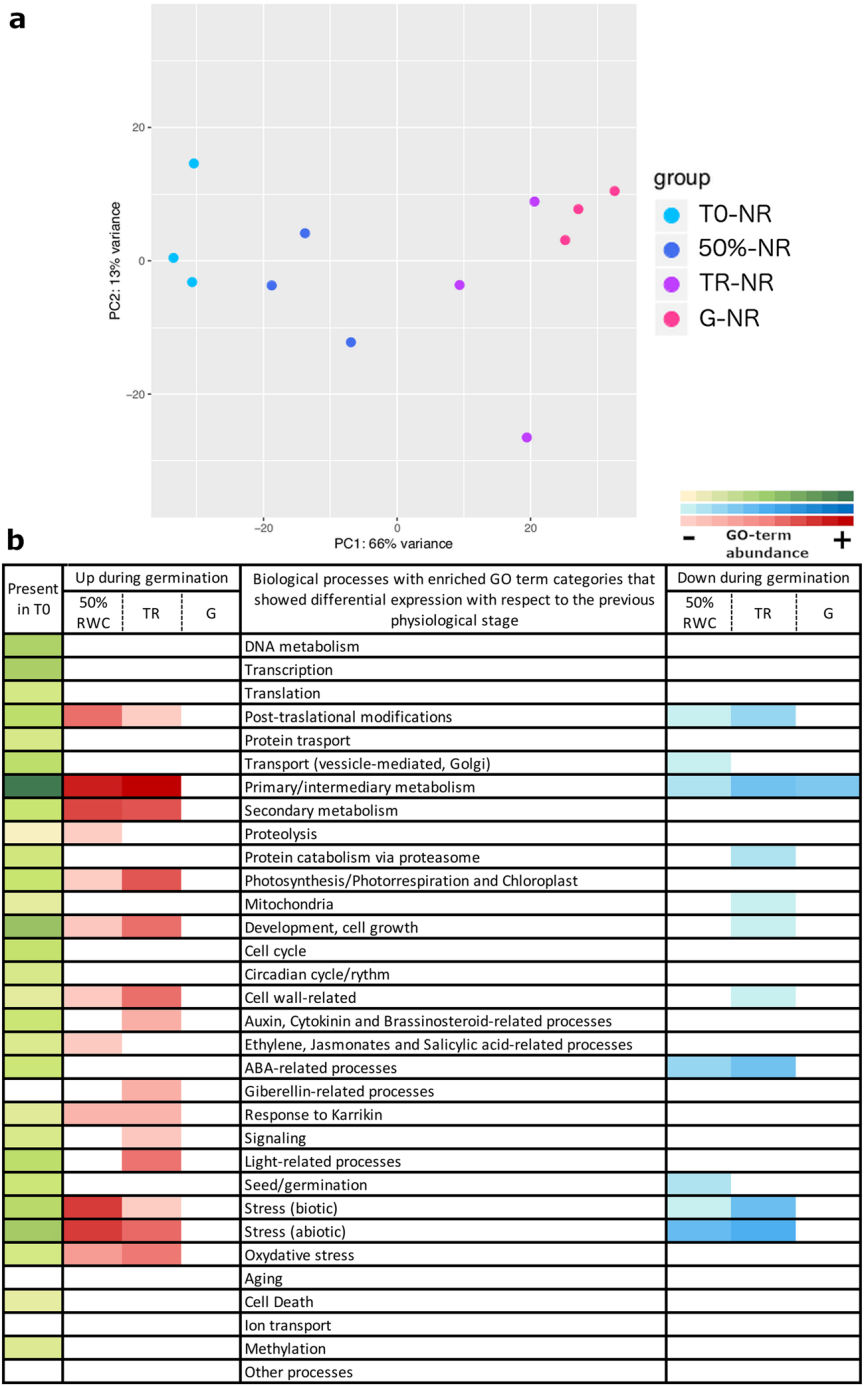
Transcriptome analyses of seed batches with negative/no response to priming. In (**a**) PCA analysis of the transcriptome profiles of three replicates for each physiological stage indicated by colour. PC1 explains 66% of the variation between the dry stage and the imbibed stages. PC2 explains 13% of the variation between replicates. In (**b**) Main GO-term categories enriched in each physiological stage. The green colour represents the enriched categories present in the dry seed while the red and blue colours represent the enriched categories that had differential expression with respect to the previous stage. Up-regulated categories are shown in red and down-regulated categories in blue.

A principal component analysis (PCA) was performed to evaluate the RWC as a physiological trait during germination (Figs. 4a and 5a). For the PR phenotype, PC1 could explain 61% of the variance between dry and imbibed seeds, and PC2 could explain 26% of the variance between the imbibed stages (16%RWC, 50%RWC, TR and G; Fig. 4a). We obtained differentially expressed genes that show significant changes between different stages and in the overall course of germination, and between physiological stages. The overall expression changes from the dry stage (T0) until radicle protrusion (G) resulted in an up-regulation of 6,635 genes and a down-regulation of 2,457 genes (Supplementary Table S2). The most important changes were detected between T0 and 16% (2,727 up-regulated and 2,375 down-regulated genes); slight changes in gene expression were detected between 16 and 50% (69 up-regulated and 26 down-regulated), but a considerable change between 50% and TR (645 up-regulated and 453 down-regulated) was also detected. A small amount of differentially expressed genes was detected between TR and G (193 up-regulated and 148 down-regulated, Supplementary Table S2). These results suggest that *C. aesculifolia* seed germination presents the early and late transcriptional phases as described for *Arabidopsis*^13^. The GO-term enrichment analyses showed that the biological groups enriched in 16%RWC with respect to T0 were those related to DNA metabolism, transcription and translation, and primary/intermediary metabolism. The response to abscisic acid (ABA), ethylene and cytokinin were also up-regulated. Abiotic stress responses, that included cadmium, cold, heat and salt stress, were upregulated. The response to ABA and abiotic stress were down-regulated. In contrast, only proteolysis related to mobilization of reserve proteins, transmembrane transport and response to water deprivation were enriched at 50%RWC and the down-regulated biological process were related to seed maturation and embryo development (Fig. 4b, Supplementary Table S3).

The enriched biological processes that represented the TR were primary/intermediary and secondary metabolism, development and cell growth, and cell wall-related processes. The response to cytokinin and auxin were also regulated as well as the response to abiotic and oxidative stresses. The main down regulated category was related to ABA. During G, the main up-regulated categories also included cell wall related processes. The primary metabolism was represented by the up-regulation of lipid metabolism and hormone response included auxin homeostasis and gibberellin response. The carbon fixation and cell cycle processes were strongly up-regulated. Enriched down-regulated biological processes were transcription, lipid storage, sucrose biosynthesis and response to ethylene and auxin.

### In the seed batches with negative or no response to priming, the dry seed transcriptome is similar to the imbibed seeds transcriptome in the early germination stages of both phenotypes

For NR phenotype, PC1 could explain 66% of the variation between the different stages (T0, 50%, TR and G) and PC2 could explain 13% between replicates (Fig. 5a). In these seed batches, expression analyses showed moderate changes between stages and in the overall course of germination, in comparison to the observed changes in PR-batches (Supplementary Fig. S5). The overall expression changes from the dry stage (T0) until radicle protrusion (G) showed an up-regulation of 2,437 genes and a down- regulation of 1,856 genes (Supplementary Table S2). In the transition from T0 to 50%RWC a total of 559 up-regulated and 247 down-regulated genes were observed, but a similar amount of expressed genes as the PR-batches between 50%RWC and TR were also detected (762 up-regulated and 470 down-regulated genes). Finally, a small amount of differentially expressed genes was detected between TR and G (21 up-regulated and 56 down-regulated, Supplementary Table S2). The biological groups that were mainly enriched in 50%RWC were those related to primary/intermediary and secondary metabolism (Fig. 5b, Supplementary Table S3). The synthesis of jasmonic acid, and biotic and abiotic stress responses were also upregulated. In contrast to PR-batches, there was not a significant up-regulation of the proteolysis category. Down-regulated biological groups were related to ABA response and embryo development.

The enriched biological processes that represented the TR were primary/intermediary and secondary metabolism, photosynthesis related processes including carbon fixation, development and cell growth and cell wall related processes. The response to light, auxin and gibberellic acid were also up-regulated as well as the response to abiotic and oxidative stresses. The main down-regulated categories were related to ABA and abiotic stress. There were also a down-regulation of biological processes related to post-translational modification and primary metabolism. During G, no significantly enriched up-regulated categories were detected. The enriched down-regulated biological process was related to lipid storage.

**Figure 6:**
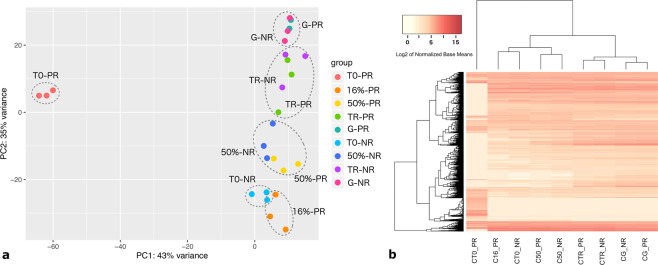
Global analysis of the transcriptome profiles of the two phenotypes during germination. In (**a**) PCA analysis of the transcriptome profiles of three replicates for each physiological stage indicated by colour. PCA was performed with the top-700 genes that accumulated the most variance across groups. PC1 indicates that the transcriptome profile of the dry stage (T0) in the PR seeds had the most important differences with respect to the other physiological stages, including the dry stage in the NR seeds (43% of variance). PC2 explains 35% of the variance between the remaining physiological stages for both phenotypes. In (**b**) heatmap of the normalized reads per transcript of all 12,683 *Arabidopsis* locus tags present in the global transcriptome assembly, and detected in at least one physiological stage. The hierarchical clustering distinguishes the two transcriptional phases^13^ indicated by low RWCs (16% and 50%) and by TR and G physiological stages. The NR-T0 (dry seed) profile is clustered among the low RWCs, been most similar to 16% transcriptomic profile.

The expression and the GO-term enrichment analyses indicated that the germination process in PR-batches occurred accordingly to what has been described in previous studies^11,13,26^ (Fig. 4b), suggesting that for *C. aesculifolia* seeds, the transcriptomic profile could be the reference for the proper transitions that arise during germination. However, in the NR-batches, the differential expression and the GO-term enrichment profiles did not reflect, at the same extent, the transitions between physiological stages. We performed time-series analyses and detected eight clusters of gene expression patterns in which the main trends in the PR batches reflected an up- or down-regulation from T0 onwards, while in the NR-batches those same clusters showed subtle or no changes from T0 onwards (Supplementary Fig. S6). These major trends from T0 to 50%RWC suggested that the transcripts levels already present in the dry seeds of the NR-batches were more similar to the observed transcript levels in imbibed seeds. To verify this observation and to test if the RWC could distinguish between physiological stages, despite the underlying differences in the phenotypes, we performed a global PCA and a heatmap with hierarchical clustering analyses (Fig. 6).

For PR and NR samples, PC1 could explain 43% of the variation between the dry and imbibed seeds except for T0-NR that was grouped with the imbibed seeds, confirming the observation from the clustered expression patterns that the NR-T0 profile is more similar to that of low RWC samples. Meanwhile, PC2 could explain 35% between the different imbibed stages and T0-NR seeds (Fig. 6a). This analysis also demonstrated that the imbibed samples of PR- and NR-batches grouped by their respective physiological stage during the germination process, including 50%RWC, TR, and G. The hierarchical clustering performed with the global transcriptomic data also confirmed that the two transcriptional phases that occur during germination are distinguishable independently of the phenotype as well as the resemblance of the NR-T0 seeds with the low RWCs seeds, by its grouping with the PR-16%RWC samples (Fig. 6b).

In *Arabidopsis*, the dry seed contains about 12,600 different mRNAs stored during development and maturation^27^. In the combined transcriptomes of both PR- and NR-dry seeds there was an overlap of about 63% (8,064) of the locus tags reported for *Arabidopsis*^27^, and about 37% (3,017) of those were shared between the two phenotypes and *Arabidopsis*. An incomplete overlap is expected due to the large differences in life histories between the two species. Like *Arabidopsis*, *C. aesculifolia* produces endospermic orthodox seeds and presents a two-stage germination, but it is a perennial tree that thrives in warm climates where water availability is the limiting factor for germination to occur. A GO-term analysis of the non-overlapping *Arabidopsis* locus tags confirmed that almost all those genes belonged to the same gene families within the biological categories presented in Figs. 4 and 5 for *C. aesculifolia*, especially stress responses, metabolism, and protein transport/modification, thus the differences between the two species is associated with their particular physiology and responses to the environment.

The results confirm that it is possible to compare seed batches of a particular species despite their differences in life histories. Using the transcriptomic profiles of PR-batches during germination as reference, we determined that the NR-dry seeds do present a transcriptomic profile corresponding to the transcriptional phase I of the germination process^13^. However, they also present some other enriched functional categories that reflect a disarray of the early transcriptional stage, like an overrepresentation of stress-related processes. The premature expression of these processes could reflect a costly and inefficient use of the seed resources, precluding the ability of NR-batches to properly respond to the priming treatment or other external stimuli.

### The NR-batches are capable of adjusting their transcriptomic profiles during the germination process, despite their initial differences with respect to the PR-batches

**Figure 7:**
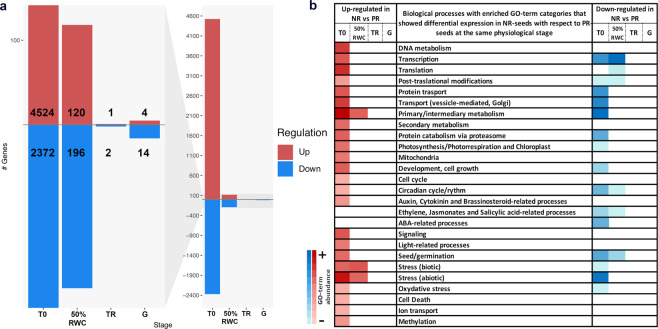
Differentially expressed genes in NR-batches with respect to PR-batches in each physiological stage. In (**a**) total gene count of differentially expressed genes in each physiological stage from the dry seed (T0) to the moment of radicle protrusion (G); the y axis represents total gene count. The left panel corresponds to a zoomed image of the graph in the right panel. In (**b**) GO-term enrichment in each category of biological processes represented in the gene set. The darker colours in the blue or red scales represents abundance of GO-terms and gene count within each category. The physiological stages TR and G had no particular enrichment of any category due to the small amount of genes detected.

The overlap of the six samples at the moment of radicle protrusion (G), and the clustering of samples by physiological stages suggested that the transcriptomic profiles of PR and NR-batches gradually become more similar during germination (Fig. 6). This is also observable in the clusters in Supplementary Fig. S6, since none of the eight clusters showed a trend in which all the genes from that particular cluster had a reversed expression trend between PR- and NR-batches (*i.e*. that the PR-batches had a trend of up-regulation at G while the NR-batches displayed a down-regulation trend at G). This trend of PR- and NR-batches to converge in transcriptomic profiles by the end of the germination process was observed even in the simulations run with a higher number of clusters or cluster membership stringency. The differential expression analyses between PR- and NR-batches for each physiological stage confirmed a reduction in the total number of differentially expressed genes during late germination (Fig. 7).

For T0 79% of the present transcripts showed differential expression, while only a 4%, 0.03% and 0.2% of transcripts present in 50%RWC, TR and G respectively were affected. In the NR-T0 seeds it was detected a GO-term enrichment in 24 of the 32 categories of biological processes in Figs. 4 and 5, of which primary/intermediary metabolism, followed by abiotic stress, translation and, DNA metabolism were the categories which presented the most abundance of terms and up-regulated genes (Fig. 7b; Supplementary Table S3). In the case of the down-regulated genes, the main categories were again the primary/intermediary metabolism as well as abiotic stress, which presented the most abundance of GO-terms and genes; but also vesicle transport, protein transport and transcription showed an important enrichment. At the 50%RWC stage, primary/intermediary metabolism, biotic and abiotic stresses were the categories that presented GO-term enrichment, while transcription was the main down-regulated category, followed by seed/germination-related processes, translation, post-translational modifications, circadian rhythm, and ethylene/jasmonic acid-related processes. For TR and G stages, no GO-term enrichment was detected. This result suggests that, although the transcriptional profile of NR-T0 is more similar to the early germination profile, these seeds are capable of adjusting their transcript levels during imbibition in order to maintain the germination program.

**Figure 8:**
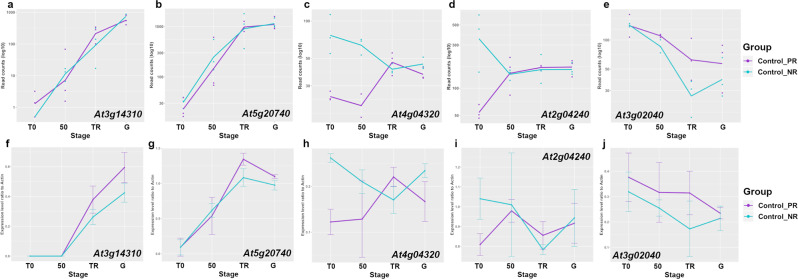
RNAseq expression profiles (**a–e**), and semi-quantitative RT-PCR validation (**f–j**) of the differential expression of five selected genes. RT-PCR data is represented as the ratio with respect to ACT7 (*At5g09810*) expression. Purple lines depict the average read counts (**a–e**) and average ratios (**f–j**) of the seed batches with positive response to priming (PR), while the turquoise lines depict the seed batches with no response/negative response to priming (NR). The dots in the upper row of panels depict the normalized read counts for each library, while the vertical bars in the lower row panels represent standard deviation of three independent PCR replicates.

Finally, transcript profiles and differential expression between PR- and NR-batches and during the germination process was verified by RT-PCR in five randomly selected genes from the data set (Fig. 8). The data was normalized using ACT7 (*At5g09810*) as reference^28^. Two of these selected genes correspond to a large gene family associated to primary cell wall modification (PME3, *At3g14310*, Fig. 8a,f; an inhibitor of PME activity *At5g20740*, Fig. 8b,g), and have been described in germination as playing important roles in testa and endosperm rupture during germination in many species^29–31^. In accordance with other studies, our RNAseq data and the RT-PCR verification showed that these two genes were consistently up-regulated in both PR- and NR- batches during germination, being at the moment of TR and G where the signal was more prominent. The gene GDPD1 (Fig. 8e,j) is associated to phospholipid metabolism, and represents the down-regulation trend in the overall gene expression during germination. No differential expression was detected between PR- and NR- batches at any stage in the RNAseq data set, and this was verified by the PCR assay. This gene has been reported in *Arabidopsis* to be down-regulated during germination, with transcript levels being undetected as soon as 3 hours after imbibition^32,33^. In *C. aesculifolia*, a similar trend was detected, although the gene is still detectable by the end of germination. The two genes that represent the differential expression between PR- and NR- batches correspond to HDH (*At4g04320*, Fig. 8c,h), involved in degradation of valine for energy production^34^, and XERICO (*At2g04240*, Fig. 8d,i), a master regulator of ABA metabolism and participates in osmotic and drought stress responses in seedlings and adult plants^35^. These two genes are up-regulated in T0 of NR-batches with respect to PR-batches, but their expression profiles converge by the end of germination. Overall, these genes reflect the main trends in expression changes detected in the data set and confirm that despite the initial differences in the dry seed between the two phenotypes, the germination program occurs in an orderly and stage-specific manner.

### Ecophysiological implications of the changes in the transcriptomic profiles of dry seeds

Even in a metabolic quiescent state such as the mature dry seed, there are a series of changes that occur at low water contents affecting diverse cellular components and macromolecules^36–38^. Seed after ripening is a process that occurs in the dry stage that can widen or increase the seeds sensitivity and perception of environmental conditions promoting germination^39^, and has been demonstrated to be a discrete developmental process^40^ that allows for reversible changes in the overall transcriptomic and proteomic profiles. This allows the embryo to adjust in response to environmental stimuli before the commitment to initiate the germination program^41^. From an ecological perspective, seed release from the mother plant in the adequate season allows for the after ripening and natural priming to occur and proper synchronization of germination with the rainy season. The PR-batches displayed a phenotype that could readily incorporate the external signal produced by the priming treatment by allowing a gradual advancement of the germination program as a fine-tuning strategy to reduce the seed-batch average time needed to complete germination once the water stimulus became stable.

In the NR-batches there are two different life histories, associated with time in storage, that converge in a loss of the capability to respond to the priming treatment and resemble an “imbibed” seed. In the 2016 seed-batch, the failure to response to priming could be a result of stressful environmental conditions experienced by the mother plants, which generated an untimely release of seeds, and a change in the transcriptome that disrupted the proper transition from seed development/maturation to the quiescent stage in which after ripening occurs^38,41^. However, for the stored batches, the seeds mRNA degrades continuously during storage^37,38^, and in a recent study, it was demonstrated that those changes can be detected even before notably changes in germination performance are perceived by standard longevity and vigour tests. These changes in the transcriptomic profile are especially notorious in longer transcripts, which can degrade faster than shorter transcripts^42^. This finding is in accordance with our rationale of studying the shifts in the transcriptome by analysing only those assembled sequences that have the features of a complete coding sequence, since the total read count would reflect only those sequences with the highest probability of still being fully functional in the seeds context. Thus, the germination capacity of the stored NR-batches in our tested conditions, along with their respective transcriptomic profiles, indicate that these seed batches might have some extent of the deleterious effects associated to storage^24,37,38^, but the transcript profiles points to a scenario of an extended and disrupted after-ripening process rather than a deleterious effect on the germination program. Overall, the advancement of the transcriptomic profile in the NR-T0 seeds could reflect a risky gamble for survival (“bet-hedging”)^43^, by decoupling the germination program from the hydric signal in order to favour germination in a wider range of conditions, but the risk of survival could be re-assigned to the next developmental phase and have cascading effects on fitness.

Phenotypic variability within and among individuals of a particular species or group of species could be a positive feature for ensuring organismal fitness and can be studied at different levels^44^. Phenology transitions are varying as climate shifts, so these changes in the timing of developmental events, important for determining organismal fitness^45^, pose another challenge to the comprehension of the basic molecular and physiological processes driving the interaction between organisms and their environment. We demonstrated that the use of physiological traits, specific to a particular developmental stage, is a reliable time-independent approach to detect common patterns between groups with wide variability and plasticity.

## Material and Methods

### Plant material

*Ceiba aesculifolia* (Kunth) Britten & Baker (*Malvaceae*) fruits were collected from 10–13 trees located within a human-activity disturbed ecosystem in the locations of “Trapiche del Rosario y Chicuasén” in Actopan Veracruz, Mexico (latitude 19.5426, longitude −96.7401, altitude 479 m a.s.l.), in February 2012, 2014 and 2015. The seed batch considered as “2016” was collected in December 2015 due to an un-timely flowering/fructification season in that year. These locations are conformed of fragments of the original tropical dry forest vegetation, accompanied by secondary and riparian vegetation, as well as several crop fields^22^. Two independent fruit batches were collected in February 2015, based on fruit morphology, which will be referred as 2015-1 (round to slightly oval) and 2015-2 (oval and elongated). The 2014 seed batch had a round to slightly oval shape as the 2015-1 batch. The fruits were transported to the laboratory, and were allowed to dry at room temperature and release the seeds. Immature or damaged seeds (*i.e*. incomplete testa formation, discoloration, or with visible cracks) were discarded. The seeds were stored in a dry room at 23–25 °C until used. For each initial batch, equal number of seeds from each mother plant was randomly selected. The 2012 and 2014 seed batches remained in storage for 5 and 1 years respectively, whereas the 2015 and 2016 seed batches were allowed a 30-day period of after-ripening to alleviate any primary dormancy remnants before any tests were performed.

### Histological study

Seeds were fixed in FAA (Formaldehyde, ethanol, acetic acid, water 2:10:1:7) for 24 h, and dehydrated in an ethanol series. Afterwards, seeds embedded in paraplast (Paraffin), and transversally and longitudinally sectioned in a rotary microtome with a 10 µm thickness. The sections were stained with Safranin-green fast^46^. The images were obtained using Olympus BX51 microscope.

### Germination performance and response to priming treatment

Seeds were submitted to a matric priming treatment. Seeds were placed in nylon mesh bags and buried in pots with a commercial soil mixture (METRO-MIX 300, Sun Gro Horticulture, MA, USA) moistened to full capacity during two days, and kept in a dark room at 23–25 °C. The upper section of the pots was covered with tin foil to avoid humidity loss. The seeds were then exhumed and allowed to dry at room temperature in darkness for two more days. *C. aesculifolia* seed germination is insensitive to light, thus imbibition in darkness does not inhibit nor promote germination in our priming conditions; however, the priming treatment was performed in total darkness in order to keep methodological consistency among seed batches collected and analysed in different years prior to the characterization of the seed response to light stimulus. Immediately after, both treated and untreated seeds were washed with 10% hypochlorite for 1 min, surface dried with a paper towel, and placed on 1% agar plates. Five replicates (30 seeds each) of all treatments were incubated in a germination chamber at 25 °C and a photoperiod 12 h:12 h light:dark. Cumulative germination was registered daily for 20 days. A seed was considered to have germinated when the radicle had protruded through the endosperm layer. The germination performance was evaluated by fitting sigmoid curves to the arcsine-transformed germination percentages obtained for each seed batch and treatment using Table Curve 2D (version 5.01.01), and final graph construction was performed in SigmaPlot (version 11.0). The following parameters were obtained from each modelled curve for statistical analysis of germination performance: germination initiation (time at which 1% of the seeds had germinated), time to 50% germination, maximum germination rate^-d^, and final germination percentage. These parameters were first tested for normality and equal variance assumptions using the Shapiro-Wilk and *F*-test respectively, and then for statistical differences between control and primed seeds per seed batch using a two-tailed Student’s t-test, at a significance level of α = 0.05. Priming response was deemed as favourable when at least one of the four parameters improved with respect to its control.

### Imbibition curves

To determine the initial seed water content (*SWC*) in each batch and treatment, 25 seeds from each batch were weighed individually and then dried in an oven for 24 h at 100 °C. The initial *SWC* was calculated individually with the following equation:

1 $$SWC\,=\,((i\,-\,d)/d)100$$

where *i* is the initial seed weight and *d* is the seed weight after drying. These SWCs were then averaged for each seed batch and treatment (*SWCa*) and used as a baseline for the initial water content. Another 15 seeds from each batch and treatment were weighed individually and then placed in 1% agar plates, which were incubated in a germination chamber at 25 °C and 12/12 h photoperiod. All seeds were continuously weighed, until radicle protrusion occurred, at 1 h intervals for the first three hours, and then progressively increasing the time interval in about five-six hours or up to 12 h as the seeds maintained a constant weight. The *RWC* was calculated with equation:

2 $$RWC=SWCa\,+\,((w\,-\,i)/i)100$$

where *SWCa* is the averaged *SWC* obtained for a particular seed batch and treatment, *i* is the initial seed weight and *w* is the imbibed seed weight. We used Table Curve 2D to select the best fitting curve that reflected the average imbibition dynamic, and each modelled curve was used to track the changes in the slope. These data was then used to generate the model curves in Fig. 3b.

### Sample collection at specific RWCs and physiological stages

Once the imbibition dynamic of each seed batch was assessed and modelled, another set of 90 seeds for control and primed seeds from each batch were individually tracked until reaching the desired RWC (16%, or 50%). These RWCs were selected based on the simplified imbibition curve generated for *C. aesculifolia* (Fig. 3b). We used the RWC ± standard deviation as the criteria for seed collection for any particular RWC at the time of measurement. The seeds exhibiting ruptured testa (TR), and germinated seeds (G) were collected and the RCW at the moment of occurrence was registered. The collection was random so at the same time of collection it was possible to collect seeds with different RWC values or different physiological states. Before the collected seeds were frozen in liquid nitrogen and stored, the testa was quickly removed and a visual inspection was performed to ensure the remainder of the seed did not show any signs of damage or decay. A total of 54 samples were stored at −80 °C until processed.

### RNA extraction and *de novo* transcriptome assembly

Total RNA was extracted from 5 seeds per sample using a modified TRIZOL protocol by Li and Trick^47^. All samples were then sent to the Unidad Universitaria de Secuenciación Masiva y Bioinformática (UUSMB), where they were processed for cDNA synthesis and Illumina HiSeq 2000 library construction. Quality assessment was performed using Qubit2.0 and quantified with Bioanalyzer 2100 prior to library construction. The libraries were sequenced and *de novo* assembled in two independent runs. In both runs, a multiplex approach was used to load the corresponding samples in the flow cells. A total of 1,165,201,460 paired-end 100pb reads were obtained from both assemblies. Illumina adaptors were trimmed using Trimmomatic and quality assessment was performed using FASTQC. Reads were also trimmed to 75pb to ensure a minimum quality score of Q20 in all bases, and low quality reads were removed. For each run, a global transcriptome was assembled using Trinity v2.1.1^48,49^. For the first assembly, 117,920 sequences were found and for the second, 398,598 sequences. Library assembly quality scores are presented in the Supplementary RNAseq data file.

### Transcript identification for functional classification

Functional annotation was determined using the Trinotate workflow to identify the main sources of contamination in each library, and the transdecoder tool to identify coding sequences of at least 100 amino acids in the 6 possible reading frames. A BLASTP was performed to select the proper reading frame for each transcript. The local base generated for this step included protein sequences from other assembled species in the *Malvaceae* family such as *Herrania umbratica*, *Durio zibethinus* and *Theobroma cacao*, and from the model plant *Arabidopsis*. We also performed a parallel filter for contaminants incorporating the organisms identified with the Trinnotate annotation report; the local base included model organisms like mouse, the *Pseudomonas aeruginosa* and the yeast pangenomes, as well as fungi such as *Trichoderma reesei*, *Fusarium fujikuroi*, *F. verticillioides* and *Diplodia corticola*. The identified proteins whose best hit matched one of the proteins in the contaminant database were filtered. We selected the best blast hit following these criteria: 60% minimal length coverage, 40% minimal identity. About 90% of confirmed transcripts matched an *Arabidopsis* locus-tag.

We then performed a cross-check validation of the Trinnotate and the transdecoder methods to confirm the identity of the transcripts and to explore the structural characteristics of the assembled transcripts. The *de novo* assembly relies on de Bruijn graphs which provide higher sensitivity for splice-variant calling, however it can overestimate transcript diversity due to assembly artefacts or transcript incompleteness^50^. Thus, after the contaminant-filtering step, the resulting transcript database included 34,274 transcripts from the first assembly and 132,271 from the second assembly, and included fully assembled sequences with open reading frames (ATG and STOP codons) flanked by possible 5′ and 3′ UTR regions, and a mixture of incomplete transcripts lacking either the 5′ or the 3′ portion of the coding sequence (no ATG or STOP codon evidence), or lacking both ATG and STOP codons. Since the ultimate purpose of the assembly relies on analysing the biologically functional processes associated to germination using *Arabidopsis* as a proxy, we decided to select only the complete transcripts and their specific read counts for posterior analyses. The complete transcript pool also included those 5′ coding sequences which were manually examined using the non-redundant protein data base in the NCBI BLAST tool to verify if they had a start codon. This filtering step ensures that the read counts used for differential expression belong to functional units, and could avoid overestimation of transcripts or biased read inflation in some locus-tags due to similarity-miscalling of sequences within gene families^50^, and takes into consideration the possibility that some of the incomplete transcripts could be a by-product of the dynamic degradation of stored mRNAs that occurs during germination^37,38,42^.

### Differential expression biological function categories analyses

We used the IDEAMEX website R-based tools for differential expression analysis, to simultaneously test differential expression with the R-workflows EdgeR, Limma, DESeq2 and NOISeq^51^. After an exploratory test, we decided to use DESeq. 2 for contrasts between physiological stages and time-series analysis using a factorial design to test the combined effects of stage and phenotype, and a reduced model to test for changes among physiological stages in each phenotype, using as cut-off values to consider a gene as differentially expressed a *P* < 0.05 and FDR < 0.05 and an expression change of at least 1 in log2 scale. The data sets of differentially expressed genes across germination were used to generate fuzzy clusters of expression patterns with the Mfuzz package, and a minimum membership threshold of 0.5. Simulations were run to test cluster stability and selected the simulation run with 8 clusters as the smallest number of clusters that reflect the major changes in the data set without excessive overlapping of clusters^52,53^. The heatmap plots were constructed with heatmap.2 (gplots v3.0.1 package) using default parameters (R v3.6.0 MacOS^54^). PCA plots were constructed with DESeq2, using the variance stabilizing transformation (vst) option for data normalization of gene counts^55^. Functional category classification was performed using the gene-ontology and KEGG-pathways options within the Functional annotation tool in the DAVID Bioinformatics Resources website (v6.8)^56–60^. The resulting GO-term and pathway clusters were then grouped in 32 main categories presented in Figs. 4b and 5b. The functional categories present in the dry seed (T0-PR and T0-NR) were ranked by abundance of GO-terms and genes contained within in order to generate the faux colour scale. The same process was performed for the up- and down-regulated categories, respectively.

### Semi-quantitative RT-PCR expression validation

A set of five randomly selected locus tags (*At2g04240*, *At3g02040, At3g14310*, *At4g04320*, and *At5g20740*), plus ACT7 (*At5g09810*, used as a control gene for normalization^29^), were used to validate RNAseq expression data. RNA samples from both the PR and NR batches at T0, 50% RWC, TR and G were used to synthesize cDNA libraries. RT-PCR runs were performed in triplicate. A set of preliminary runs of 20, 25, 30, 32 and 35 cycles were performed in order to verify the best amplification conditions. At 32 cycles, ACT7 and *At2g04240* showed saturation, thus all runs were performed at 30 cycles. The primers used are shown in Supplementary information Table S4. Images with the PCR products were processed in GIMP (2.10, MACOS) and data was imported into excel and R for graph construction.

## Supplementary information

Supplementary Information.

Supplementary Information 2.

Supplementary Information 3.

Supplementary Information 4.

## Data Availability

Reads were deposited in the SRA platform within the NCBI GenBank website under Bioproject PRJNA561202, and accession numbers SAMN12611291 and SAMN12611291. Assembled sequences were deposited in the TSA platform under accession number GHVB00000000.
