# Supplementary material for: The seed water content as a time-independent physiological trait during germination in wild tree species such as Ceiba aesculifolia
Source: Sci Rep. 2020 Jun 26;10:10429. doi: 10.1038/s41598-020-66759-3 (PMC7319967; doi:10.1038/s41598-020-66759-3)
Supplement: Supplementary file 1 — Supplementary Information. [file 41598_2020_66759_MOESM1_ESM.pdf]

Supplementary figures and table for the manuscript: **The seed water content as a time-independent physiological trait during germination in wild tree species such as *Ceiba aesculifolia***, by Gómez-Maqueo, X., Soriano, D., Velázquez-Rosas, N., Alvarado-López, S., Jiménez-Durán, K., Garciadiego, M., and Gamboa-deBuen, A.

Fig. S1. Climograph of the "Trapiche del Rosario y Chicuasén" locality within Actopan, Veracruz (Mexico). Blue bars depict the mean precipitation (mm) per month. The red, orange and yellow lines depict the maximum, mean and minimum average temperatures per month (ºC), respectively. The arrow above February indicates the moment at which *C. aesculifolia* seeds as shed on a regular season.

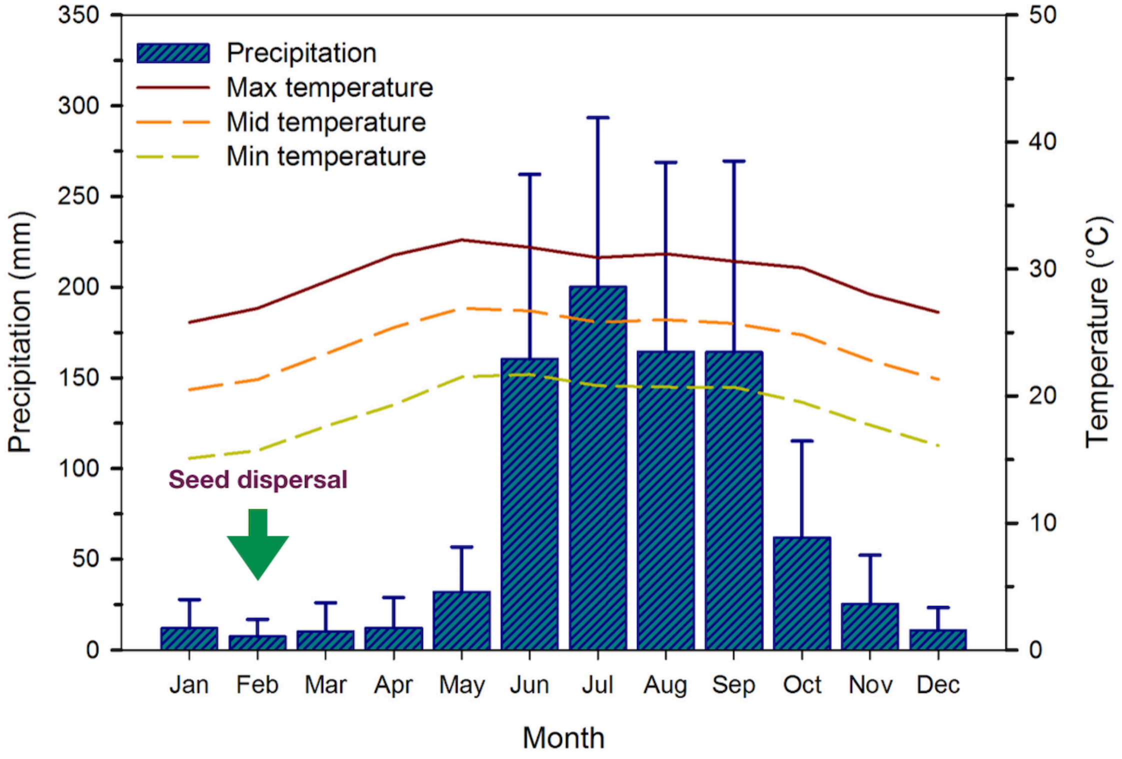

Fig. S2. Seed germination performance of seed lots with positive (2014, 2015-1) and negative (2012-5y, 2016) response to priming. In all graphs, letters denote statistical differences in the start of germination(near the X axis), time to 50% (middle), and final germination (top right corner). The asterisk (*) in the 2016 graph indicates a statistically significant decrease in the maximum germination rate in primed seeds with respect to control seeds (see table 1).

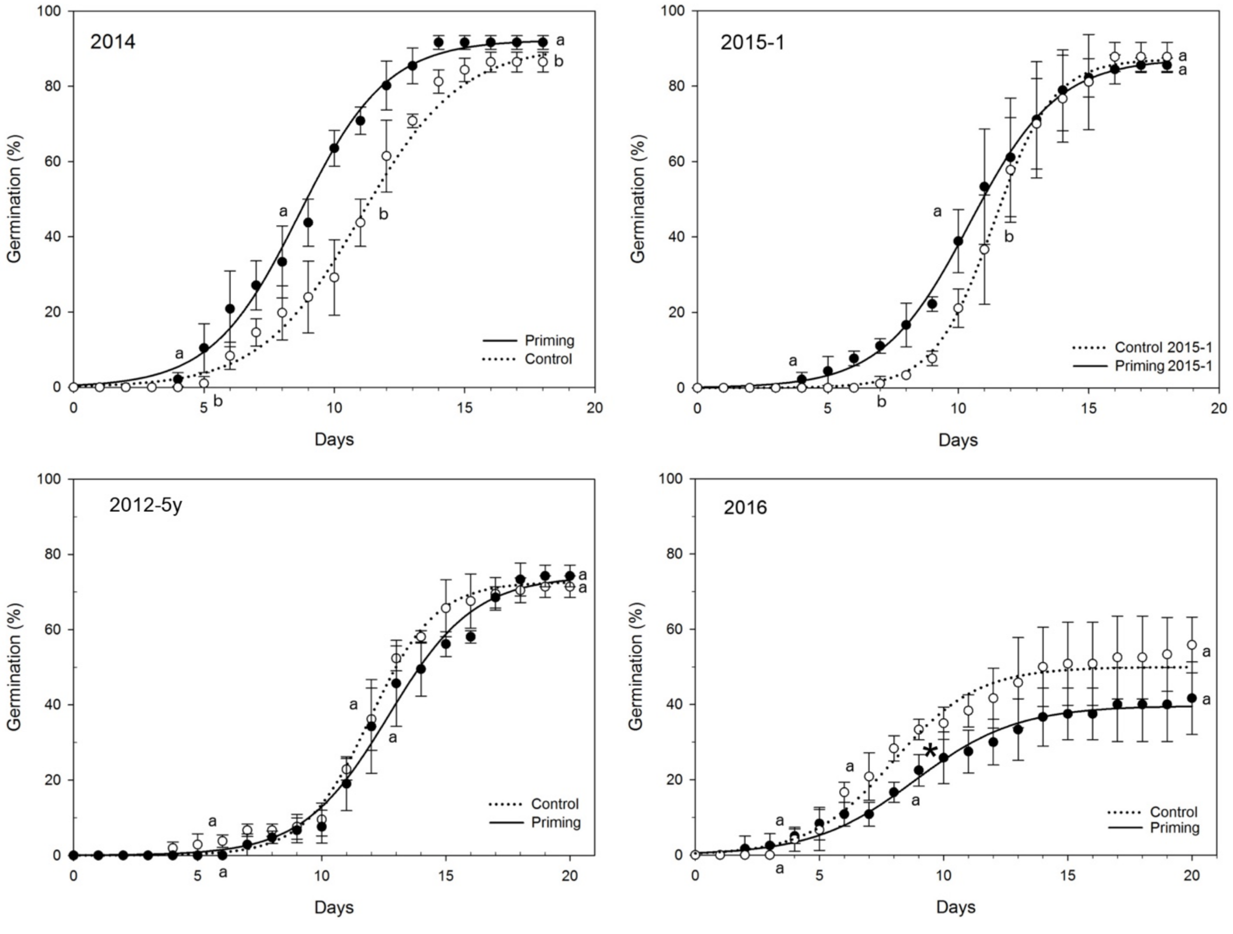

Table S1. Summarized germination performance of the six seed batches analysed, based on four germination features: germination initiation, maximum germination rate, day to attain 50% germination, and final germination. The numbers in bold denote significant differences for a two-tailed *t* test with 8 degrees of freedom and α=0.05 (*P*-values in parenthesis) of primed seeds in comparison to its respective control, for each seed batch. Results are presented as the average of 5 independent replicates and its respective standard deviation. The last column denotes the decision to consider a particular seed batch as having a positive response to priming (PR), or non-responsive to priming (NR), based on the germination test. The numbers with an asterisk (*) in each priming row denote those comparisons for which a Mann-Whitney test was performed due to a failed normality test at an α=0.05 threshold.

Seed Batch	Treatment	Germination initiation (day)	Maximum rate (germination %·d^{-1})	Day to 50% germination	Final germination %	Priming phenotype
2014	Control	5 ± 0.7	8.95 ± 0.68	11.6 ± 0.37	86.45 ± 2.6	PR
2014	Priming	4 ±0.61 (0.08)	**10.65 ± 1.1** (0.02)	**9.3 ± 0.89** (0.001)	**92.33 ± 3.34** (0.03)	PR
2015-1	Control	7 ±1.17	12.4 ± 3.3	12.24 ± 1	87.78 ± 3.85	PR
2015-1	Priming	**4 ± 1.05** (0.01)	**7.96 ± 2.14** (0.03)	11.7 ± 1.24 (0.6)	85.56 ± 1.92 (0.34)	PR
2015-2	Control	6 ± 0.48	13.08 ± 0.24	10.42 ± 0.46	87.78 ± 1.92	PR
2015-2	Priming	**4 ±1** (0.01)	14.69 ± 3.06* (0.31)	**8.55 ± 0.48** (0.01)	90.0 ± 3.33 (0.37)	PR
2014-3y	Control	5 ± 0.6	6.99 ± 1.62	12.93 ± 1.17	94.0 ± 2.79	NR
2014-3y	Priming	6 ± 0.81* (0.91)	7.3 ± 1.34 (0.75)	14.09 ± 2.19 (0.33)	85.33 ± 8.69 (0.07)	NR
2012-5y	Control	5 ± 1.46	6.48 ± 1.29	10.94 ± 0.4	71.43 ± 2.86	NR
2012-5y	Priming	6 ± 0.84 (0.12)	7.49 ± 0.49 (0.27)	11.52 ± 1.08 (0.43)	74.29 ± 2.86 (0.14)	NR
2016	Control	4 ± 2.79	8.05 ± 0.984	6.26 ± 1.22	53.33 ± 8.5	NR
2016	Priming	2 ± 3.65* (0.07)	**3.94 ± 0.63** (0.001)	6.77 ± 0.69 (0.5)	46.0 ± 12.78* (0.11)	NR

Fig. S3. Seed imbibition variability per hour in seed batches with positive response to priming. The imbibition axis is shown as a category rather than a continuous scale to facilitate interpretation.

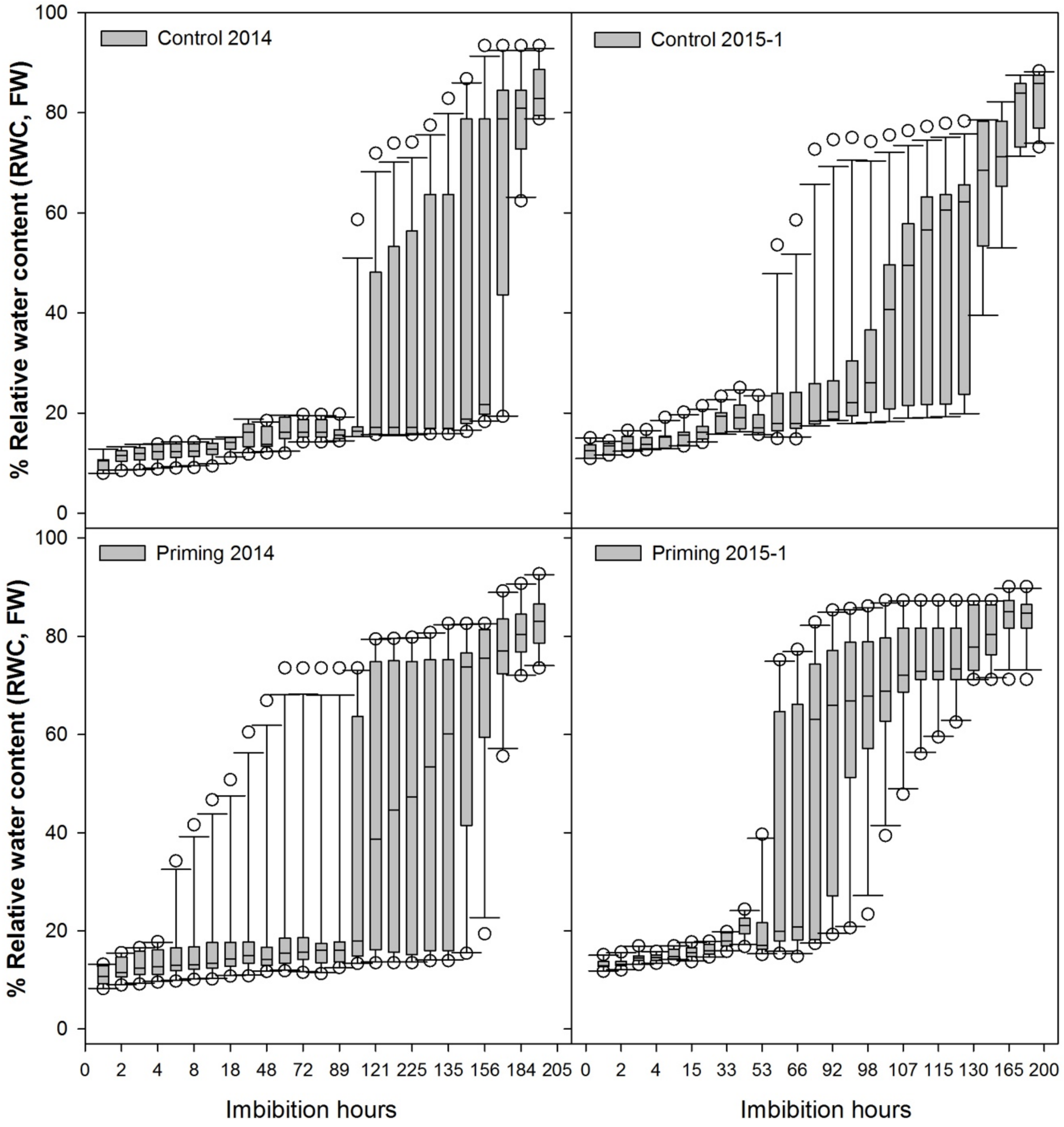

Fig. S4. Seed imbibition variability per hour in seed batches with negative response to priming. The imbibition axis is shown as a category rather than a continuous scale to facilitate interpretation.

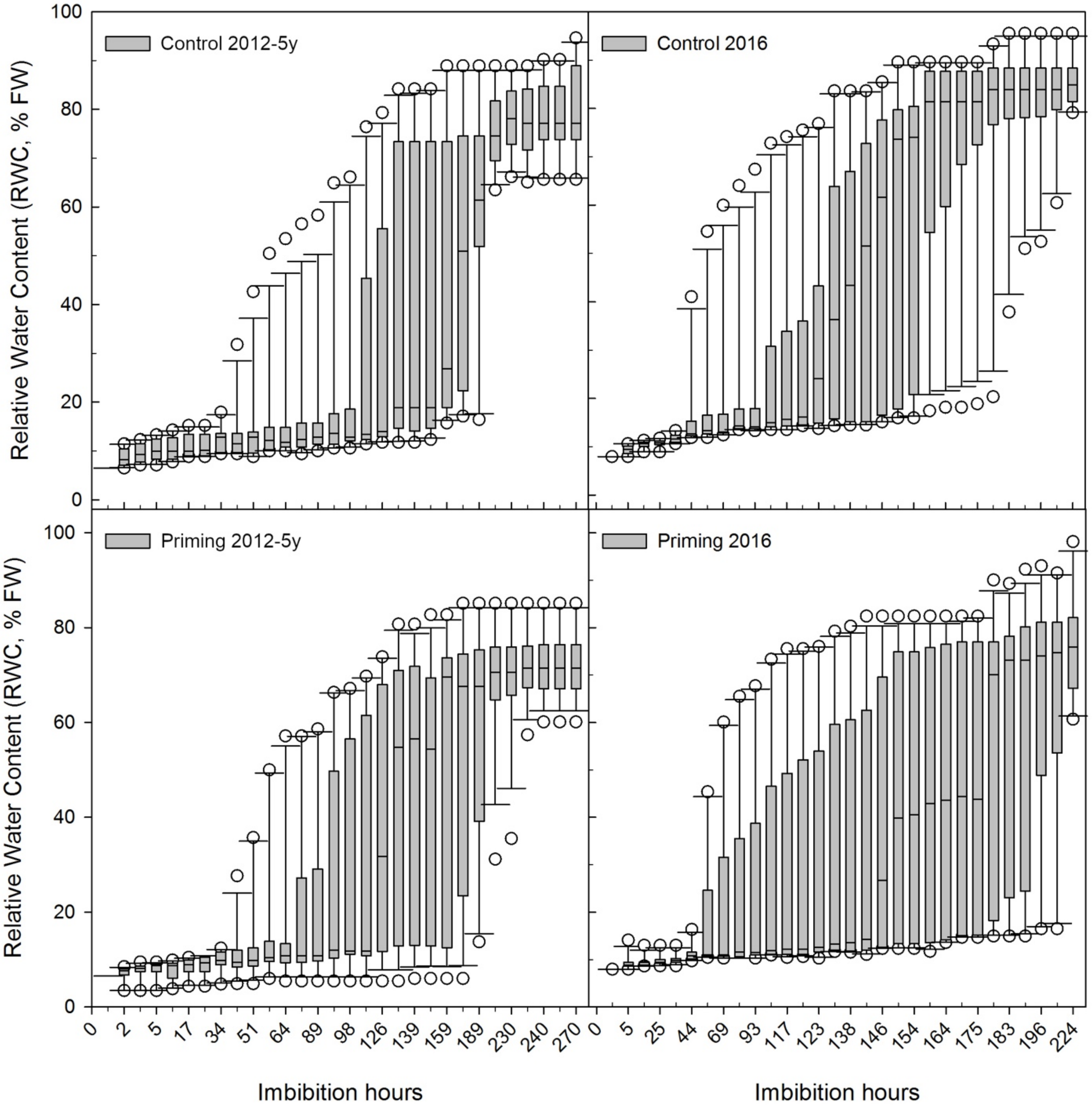

Fig. S5. Venn diagrams of the total number of genes differentially expressed in the two phenotypes. A) Venn diagram of the up- or down-regulated genes in at least one physiological stage during germination in each phenotype. Ellipses from left to right: up-regulated genes in the PR-batches, down-regulated genes in PR-batches, up-regulated genes in the NR-batches, and down-regulated genes in NR-batches. B) Comparison between the regulated genes (up- and down-regulated) in each phenotype and the overall differential expression pattern during of the NR-phenotype during germination in relation to the observed expression pattern in the PR-phenotype (*i.e.* the genes that on average had higher or lower read counts in NR in comparison to PR over the whole duration of the germination process).

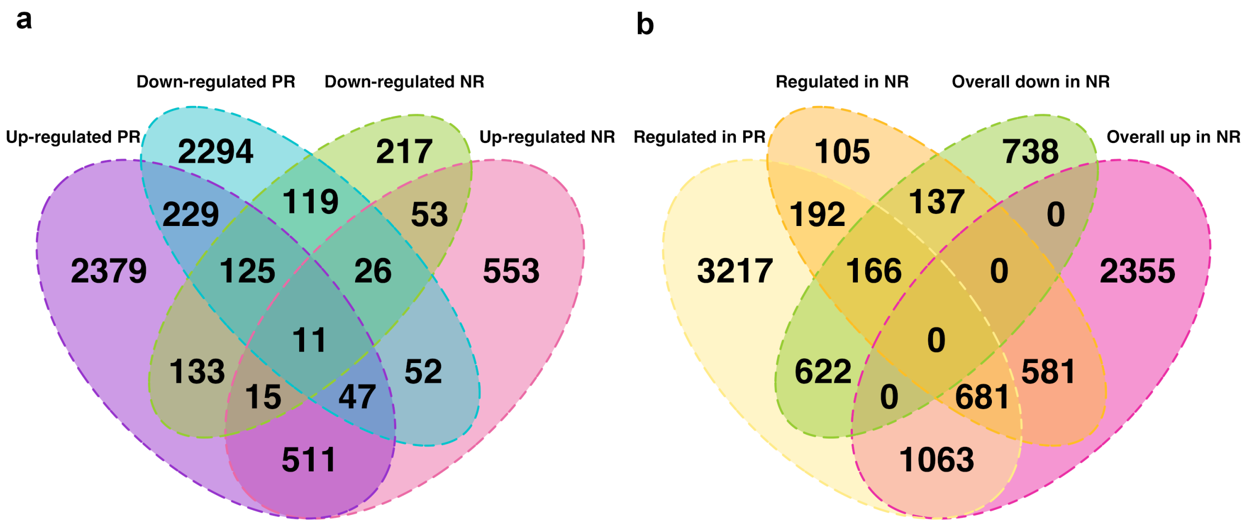

Fig. S6. Time-series clusters of the main gene expression patterns detected in the differentially expressed genes above a threshold of 0.35 of overall difference during germination between PR- and NR-batches. The total number of genes in each cluster are shown. From a to d, gene clusters that had an overall up-regulation in PR batches (purple clusters), and in NR-batches (turquoise batches). In a and d is notoriously different the average read counts at T0 in NR-batches. In e and f are the clusters with a down-regulation trend during germination, while in g and h the main pattern of expression changes by the 50% stage in PR- batches, but in NR-batches that trend is not notorious due to a total read count in T0 distinctively above or below the observed read counts in PR-batches.

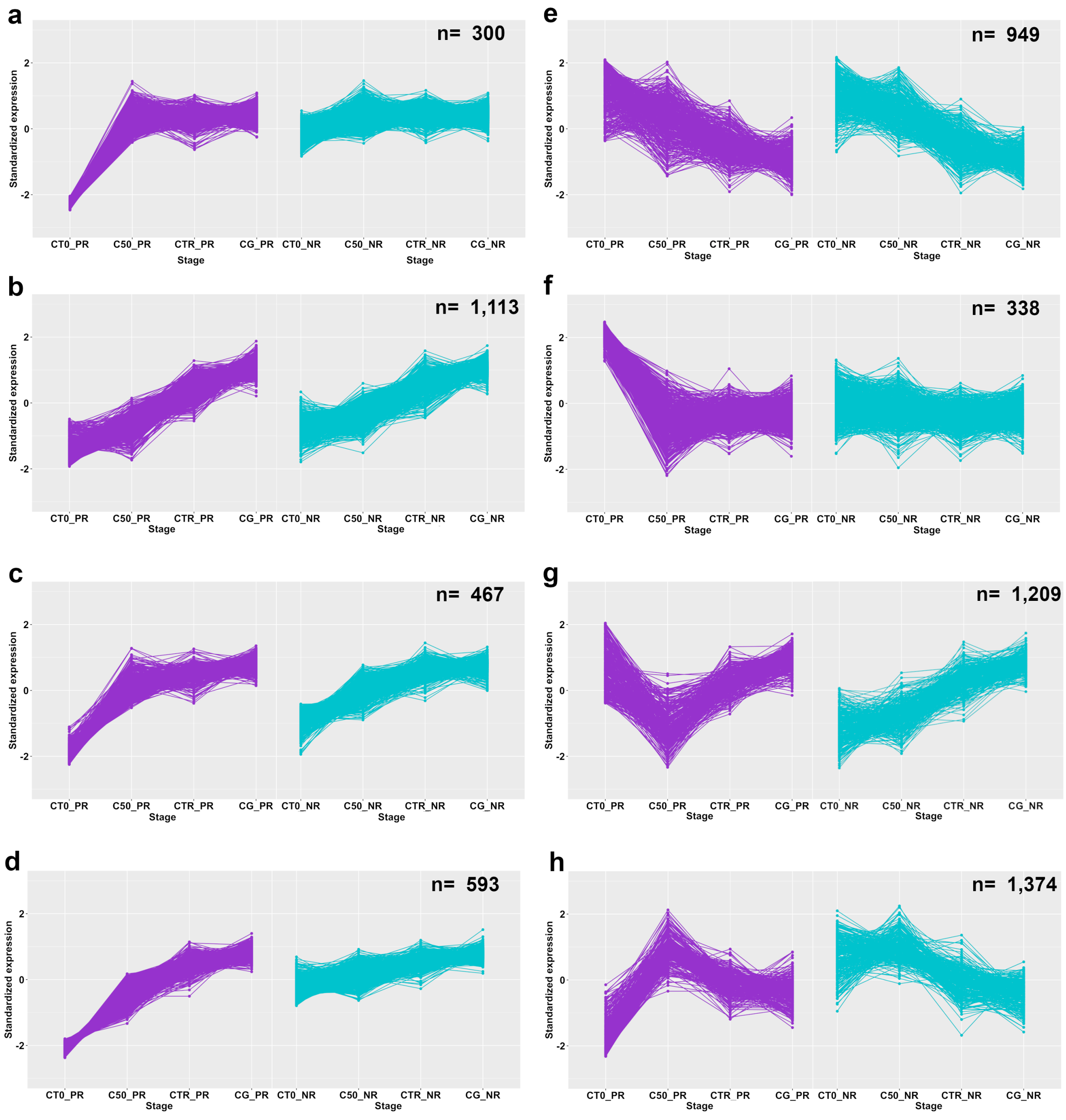

Table S4. Primers of the selected genes used for the semi-quantitative RT-PCR validation of RNAseq expression data.

**Gene name**	**Locus tag**	**Primer sequence (5´- 3’)**	**PCR product size (pb)**	**Tm (°C)**	**%GC**
Actin 7	*At5g09810*	F- CTGCCATGTATGTTGCCATC<br>R- ACGGAATCTCTCAGCTCCAA	380	59.96<br>59.95	50.00<br>50.00
PME3	*At3g14310*	F -ATCAACGGTTCCTGCTACGA<br>R - CAGTTGCTGTGGTTGGTGAA	209	59.11<br>58.90	50.00<br>50.00
PMEI-like	*At5g20740*	F -GTCAAAGTCAGCCTGTCACG<br>R -CCCCACTCACCCATGTCTG	315	59.14<br>59.70	55.00<br>63.16
MCD	*At4g04320*	F - GGAGTTGGTCGTCGTTGTTT<br>R - CCAGTAGCAAGATCCCCAGA	464	60.01<br>60.21	50.00<br>55.00
GDPD1	*At3g02040*	F - CAGGGCAGCATCGTTTAAGT<br>R - TGGATGAGATGGTCTTGCTG	492	60.27<br>59.79	50.00<br>50.00
Xerico	*At2g04240*	F - CGTCGTTATCACCATCCCCT<br>R -ACCACTTTTCGAGGCACACC	249	59.25<br>60.82	55.00<br>55.00
